# Comparing the Biofilm Removal Capacity of NaOCl, Povidone-Iodine, Chlorhexidine, Curcumin, and Triphala as Endodontic Irrigants

**DOI:** 10.7759/cureus.52067

**Published:** 2024-01-10

**Authors:** Nuha Alghamdi, Bhaskar Das, Santosh I Hugar, Priyanka Sarangi, Garima Garg, Savadamoorthi Kamatchi Subramani

**Affiliations:** 1 Department of Restorative Dental Sciences, College of Dentistry, King Khalid University, Abha, SAU; 2 Department of Dentistry, Diphu Medical College and Hospital, Diphu, IND; 3 Department of Conservative Dentistry and Endodontics, Vasantdada Patil Dental College and Hospital, Sangli, IND; 4 Department of Conservative Dentistry and Endodontics, Sriram Chandra Bhanja (SCB) Dental College and Hospital, Cuttack, IND; 5 Department of Conservative Dentistry and Endodontics, Government Dental College and Hospital, Nagpur, IND; 6 Department of Conservative Dentistry and Endodontics, College of Dentistry, Jazan University, Jazan, SAU

**Keywords:** root canal, biofilm, triphala, curcumin, chlorhexidine, povidone iodine, naocl

## Abstract

Background and aim: A sessile multicellular organism that is immersed in a self-produced matrix of extracellular polymeric substances and has its cells firmly attached to a surface is referred to as a microbial biofilm. When it comes to pulp and periradicular pathosis, biofilms are crucial. To reduce the number of microorganisms in the root canal and assist in treating periapical pathosis, endodontic therapy must include decontamination of the system of tooth root canals through biomechanical preparation and irrigation of the root canal. This study compares sodium hypochlorite (NaOCl), povidone-iodine, chlorhexidine, curcumin, and triphala as endodontic irrigating solutions regarding their capacity to eliminate biofilm from root canals.

Materials and methods: A total of 60 patients were included if they had pulpitis. Two specific samples (samples A and B) were chosen for analysis from a collection of samples so that their bacterial composition is most similar to that of acute pulpitis. The suspensions of bacterial cells from this polymicrobial culture have been collected from frozen stock and then regrown by inoculation on Columbia agar base (Basingstoke, UK) with the addition of vitamin K1, hemin, and 5% (v/v) calf blood. The pureness of the suspensions was assessed using colony morphology and Gram staining. Analytical profile index (API) 20A tests or automated test for bacteria (ATB) ID 32A tests were initially used to identify the isolates. These polymicrobial cultures' in vitro biofilms were developed using membrane filters made of cellulose nitrate. The tested irrigating solutions were as follows: 5.25% sodium hypochlorite, 10% triphala, 0.2% chlorhexidine gluconate, 10% povidone-iodine, and 5% curcumin (CUR). On the other hand, phosphate-buffered saline was taken as a control agent.

Results: As the standard of excellence in endodontic irrigation, NaOCl has eliminated all germs in sample A following 15 minutes of culture and in both of the specimens after 40 minutes. Iodine also eliminated all germs after 40 minutes of administration, indicating that it would be worth exploring using iodine as a potential endodontic irrigant. Iodine achieved total bacterial elimination after 40 minutes in both samples; however, it was less effective after 15 minutes. Our findings indicate that iodine solution is the most suitable alternative after the supremely effective NaOCl, although it requires longer contact times to generate the necessary and recognized broad-spectrum antibacterial properties, including in the case of biofilms. Furthermore, curcumin also showed significant results after NaOCl and iodine.

Conclusion: The antibacterial potency of each studied irrigant was significant, supporting their usage in endodontics. It was observed that NaOCl has the maximum antibacterial activity.

## Introduction

Endodontic therapy must include an important step for the removal of microorganisms from the tooth's root canal. Clinicians should have sufficient knowledge and expertise in this important aspect of endodontics [1,2]. The eradication of microorganisms from the system of root canals, the elimination of infected pulpal tissue, and the possibility of preventing recontamination are all goals of endodontic therapy [3,4]. To reduce the number of microorganisms in the root canal and assist in treating periapical illness, endodontic therapy must include decontamination of the tooth root canal system through biomechanical preparation and irrigation of the root canal [5]. The aforementioned agents have no antimicrobial effect and will not considerably lower the bacterial burden [6]. Such irrigation agents can be used regularly because they are accessible and simple to use. Local anesthetic solutions are likewise packaged uncontaminated and are simple to administer using extremely small-diameter needles [7,8]. Safety may be another aspect that influences their utilization. The management of root canals that are infected shouldn't use these irrigants. Several more effective irrigation options are more suitable for treating infected root canals [9,10]. The most commonly suggested and employed endodontic irrigating solution is sodium hypochlorite (NaOCl). It has both pulpal disintegration and an antibacterial action as benefits. Strongly alkaline sodium hypochlorite (NaOCl) (pH > 11) functions as a chemical solvent to degrade and hydrolyze amino acids by generating chloramine molecules. There is empirical evidence supporting that when NaOCl is used for endodontic therapy in teeth suffering from apical periodontitis, bacterial populations can be reduced [5-11].

Iodine was first used in endodontic therapy in 1979, and povidone-iodine was encouraged because it was non-allergenic, effective against a broad spectrum of bacteria, relatively harmless, and less likely to discolor dentine [7,8]. Iodine has since been demonstrated to have sporicidal, virucidal, tuberculocidal, fungicidal, and bactericidal properties. Two percent formulations of iodine potassium iodide (IPI), which are used as endodontic therapy, have been proven to be less harmful and irritant than cresatin, camphorated monochlorophenol (FMCP), and formocresol. This gives iodine an edge over such irritants [9,10].

An antimicrobial made of cationic bis-biguanides is chlorhexidine (CHX). Its benefits are dependent on a wide range of activities. Resistance is not as probable since CHX targets numerous locations at the cellular level [12,13]. In the membranes of cells, lipopolysaccharides as well as phospholipids engage with CHX, a positively charged hydrophilic and lipophilic molecule. As a result, the cell membranes are damaged, allowing CHX molecules to get inside the cell and generate intracellularly harmful consequences such as cytoplasmic coagulation [14,15]. With greater effectiveness against Gram-positive bacteria, CHX is bacteriostatic at reduced concentrations and bactericidal at greater concentrations against both Gram-positive and Gram-negative germs [15].

Since ancient times, people have utilized *Curcuma longa* (turmeric), a member of the Zingiberaceae family, as a kind of traditional medical treatment [13]. The primary yellow therapeutic component of turmeric, curcumin (diferuloylmethane), has been shown to have antiviral, antiprotozoal, antifungal, antibacterial, antioxidant, and anti-inflammatory properties. The elimination of *Enterococcus faecalis*, one of the prevalent bacteria associated with failure of root canal therapy, by *C. longa* demonstrated promising outcomes [14]. *C. longa* could serve as a suitable, affordable replacement for all previously used intracanal medications because it has fewer negative effects and produces the least amount of bacterial resistance of the species [15].

Three medicinal plants (*Emblica officinalis*, *Terminalia chebula*, and *Terminalia bellerica*) produce dried, crushed fruits that make up the ayurvedic herbal preparation known as triphala. Tannic acid is the main component in it [16]. It has been traditionally used in Indian medicine to treat liver problems, headaches, and constipation. Tannic acid has an inhibitory effect on the growth of bacteria as well as bactericidal effect on Gram-positive as well as Gram-negative bacteria, according to early investigations [17]. Triphala has several benefits, including simplicity of accessibility, affordability, durability over time, minimal toxicity, and lack of germ resilience. Triphala is harmless and has additional anti-inflammatory qualities and antioxidant capabilities. It is made up of substances with adequate physiologic actions when compared to numerous regularly used root canal irrigants [18,19].

To the best of the author's knowledge, there has been no study in the past that has compared the above-mentioned root canal irrigants in eliminating biofilms from root canals. This study compares NaOCl, povidone-iodine, chlorhexidine, curcumin, and triphala as endodontic irrigating solutions regarding their capacity to eliminate biofilm from root canals.

## Materials and methods

Specimens were obtained as part of continuous root canal therapy; 60 patients were included only if they had necrotic pulp. The sample size was calculated using the following formula: n=Z^2^×p(1−p)/E^2^, where n is required sample size, Z is z score corresponding to the desired confidence level (e.g., 1.96 for 95% confidence), p is the estimated proportion of the population, and E is margin of error (desired precision). Two particular samples, denoted as samples A and B, were deliberately selected from a pool of specimens to closely resemble the bacterial composition typically associated with acute pulpitis. With proper sterilization, an endodontic procedure was carried out. Supragingival biofilms were removed from each tooth using pumice scaling and cleaning before rubber dam isolation. Utilizing round burs as well as Endo-Z burs (Ballaigues, Switzerland: Dentsply Maillefer), traditional access cavities were produced. Dental floss was firmly wound around the tooth's neck after the rubber dam had been applied.

The next step was a thorough disinfection with 2.5% NaOCl wiping of all surfaces. The operating area, comprising the pulp chamber, was additionally cleansed and decontaminated with 2.5% NaOCl, while the access preparation was completed with a second sterile bur under being irrigated with a solution of sterile saline. The root canal space was gently washed with 1 mL of a sterile water solution to eliminate detached cells, and the specimen was collected by the consecutive application of three to five sanitized paper points (Dentsply, India), according to the procedure by Siqueira and Rôças [20]. For one minute, every single paper point stayed in the canal. These paper points were transferred to anaerobic transport media and then immediately processed at the Department of Microbiology. For this experiment, two polymicrobial cultures (samples A and B) were selected from teeth with a clinical diagnosis of pulpitis consisting of a root canal infection with viable pulp. Alpha-hemolytic Streptococcus sp., *Hafnia alvei*, *Bacteroides ureolyticus*, *Actinomyces odontolyticus*, and *Actinomyces meyeri* bacteria were present in sample A. Alpha-hemolytic Streptococcus sp. strains, *Finegoldia magna*, *A. meyeri*, and *Parvimonas micra* were present in sample B.

The suspensions of bacterial cells from this polymicrobial culture have been collected from frozen stock and then regrown by inoculation on Columbia agar base (Basingstoke, UK) with the addition of vitamin K1, hemin, and 5% (v/v) calf blood. The pureness of the suspensions was assessed using colony morphology and Gram staining. Analytical profile index (API) 20A tests or automated test for bacteria (ATB) ID 32A tests were initially used to identify the isolates. These polymicrobial cultures' in vitro biofilms were developed using membrane filters made of cellulose nitrate. The dimensions of these membrane filters were 13 mm in diameter and 0.2 mm in pore size. They contained 5x10^8^ colony-forming units (CFU)/mL (colony-forming unit) of every bacterial type. Further, 20 µl of broth with dispersion of bacteria in infusion broth of the brain and heart having pH 7.2 (Oxoid, UK) was used for inoculation of the cellulose nitrose membranes after they had been set over the outer layer of enriched blood agar. The plates, each containing a single membrane filter, were assembled and incubated for two days at a temperature of 37°C in anaerobic conditions with an atmosphere composed of 5% CO_2_ (carbon dioxide), 5% H_2_ (hydrogen), and 90% N_2_ (nitrogen).

After the incubation was complete, the membrane filters were carefully taken out from the agar plates and placed into 5 mL solutions of the chosen endodontic antimicrobial irrigating solution or a control solution, where they were incubated for 15 minutes and 40 minutes at room temperature without mechanically disrupting the biofilm. The tested irrigating solutions were as follows: 5.25% sodium hypochlorite, 10% triphala, 0.2% chlorhexidine gluconate, 10% povidone-iodine, and 5% curcumin (CUR). On the other hand, phosphate-buffered saline (PBS) was taken as a control agent.

The membrane filters were cautiously withdrawn from the provided suspensions following the period of incubation and placed in neutralizing broth (PBS) with a pH of 7.2 for 5 minutes. Each of these suspensions was then diluted (10-1-10) in BHI (Brain heart infusion) broth after being shaken on a vortex shaker for 60 seconds. In the first step of the dilution process, a small amount (usually 1 part) of the original sample is mixed with 9 parts of a diluent (BHI broth). This results in a 10-fold dilution. In the second step, a small amount (usually 1 part) of the first diluted solution is taken and mixed with 9 parts of the diluent again. This results in an additional 10-fold dilution, for a total reduction of 100-fold from the original concentration. On non-selective media, 100 µl aliquots of all dilutions and 100 µl samples of the corresponding original suspension were plated. The amount of growing facultative as well as obligate anaerobic bacterial flora was measured using a Columbia agar base enriched with vitamin K1 (1 g/mL), hemin (5 g/mL), and 5% (v/v) of blood. The culture plates were incubated for four days at a temperature of 37°C in an anaerobic setting with an atmosphere composed of 5% CO_2_, 5% H_2_, and 90% N_2_. Following the completion of the incubation period, the number of colony-forming units (CFU) in every disc was determined by counting each of the several colony types. To calculate an average, each measurement was made three times. The actual therapeutic agents were hidden from the examiners.

Statistical analysis

Statistical analyses were carried out in Statistica for Windows 11.0 (Tulsa, OK: StatSoft, Inc.). Analysis of variance (ANOVA) was used for a statistical assessment of the comparison of efficacies with PBS, and Tukey's honestly significant difference (HSD) was used as the post-hoc test.

## Results

Sample A

Fifteen-Minute Incubation

The mean CFU in specimens treated with PBS solution (control) was 350800011±114. The mean CFU in specimens treated with iodine endodontic irrigating solution was 40021±113. The CFU count was reduced significantly in comparison to the PBS control (p=0.000152). The mean CFU in specimens treated with NaOCl endodontic irrigating solution was 0, showing complete elimination. The CFU count was reduced significantly in comparison to the PBS control (p=0.000198). The mean CFU in specimens treated with CUR endodontic irrigating solution was 6677±216. The CFU count was reduced significantly in comparison to the PBS control (p=0.000126). The mean CFU in specimens treated with CHX endodontic irrigating solution was 4283344±182. The CFU count was reduced significantly in comparison to the PBS control (p=0.000167). The mean CFU in specimens treated with a triphala endodontic irrigating solution was 6671±152. The CFU count was reduced significantly in comparison to the PBS control (p=0.000105). In terms of maximum CFU count reduction against the biofilm, the order of antibacterial activity observed was as follows: sodium hypochlorite (NaOCl)>iodine irrigant solution>triphala. The reduction in CFU in CUR (6577±216) and triphala (6671±152) was comparable, with almost similar antibacterial activity against biofilm. A minimum reduction was observed in CHX-irrigating solutions showing the least antibacterial activity. The antibacterial activity was in the following sequence: NaOCl>iodine>CUR=triphala CHX>PBS.

Forty-Minute Incubation

The mean CFU in specimens treated with PBS solution (control) was 359050021±132. The mean CFU in specimens treated with an iodine endodontic irrigating solution was 0. The CFU count was reduced significantly in comparison to the PBS control (p=0.000219). The mean CFU in specimens treated with NaOCl endodontic irrigating solution was 0, showing complete elimination of bacteria. The CFU count was reduced significantly in comparison to the PBS control (p=0.000238). The mean CFU in specimens treated with CUR endodontic irrigating solution was 97±15. The CFU count was reduced significantly in comparison to the PBS control (p=0.000106). The mean CFU in specimens treated with CHX endodontic irrigating solution was 78875±132. The CFU count was reduced significantly in comparison to the PBS control (p=0.000200). The mean CFU in specimens treated with triphala endodontic irrigating solution was 6689±114. The CFU count was reduced significantly in comparison to the PBS control (p=0.000211). It was observed that a maximum reduction in CFU count was observed in NaOCl-treated specimens, showing maximum antibacterial activity against biofilm. CUR and iodine came after them. The reduction in iodine and CUR was comparable, with almost similar antibacterial activity against biofilm. It was followed by a triphala irrigation solution. A minimum reduction was observed in CHX-irrigating solutions showing the least antibacterial activity. The antibacterial activity was in the following sequence: NaOCl>iodine>CUR>triphala>CHX>PBS (Table 1).

**Table 1:** Comparison of the effectiveness of various irrigation methods in sample A with PBS as the control. CFU: colony-forming units; SD: standard deviation; PBS: phosphate-buffered saline; CUR: curcumin; CHX: chlorhexidine; NaOCl: sodium hypochlorite

Variables	15 minutes (mean CFU± SD)	p-Value	40 minutes (mean CFU±SD)	p-Value
Iodine	40021±113	0.000152	0	0.000219
NaOCl	0	0.000198	0	0.000238
CUR	6577±216	0.000126	97±15	0.000106
CHX	4283344 ±182	0.000167	78875±132	0.000200
Triphala	6671±152	0.000105	6689±114	0.000211
PBS	350800011±114	0.0001	359050021±132	0.0001

Sample B

Fifteen-Minute Incubation

The mean CFU in specimens treated with PBS solution (control) was 188531111±121. The mean CFU in specimens treated with iodine endodontic irrigating solution was 380022±113. The CFU count was reduced significantly in comparison to the PBS control (p=0.000311). The mean CFU in specimens treated with NaOCl endodontic irrigating solution was 16671±108. The CFU count was reduced significantly in comparison to the PBS control (p=0.000931). The mean CFU in specimens treated with CUR endodontic irrigating solution was 360031±312. The CFU count was reduced significantly in comparison to the PBS control (p=0.000342). The mean CFU in specimens treated with CHX endodontic irrigating solution was 18561143±416. The CFU count was reduced significantly in comparison to the PBS control (p=0.000363). The mean CFU in specimens treated with triphala endodontic irrigating solution was 255555±142. The CFU count was reduced significantly in comparison to the PBS control (p=0.002924). It was observed that a maximum reduction in CFU count was observed in NaOCl-treated specimens, showing maximum antibacterial activity against biofilm. They were followed by iodine. CUR and triphala came after it. A minimum reduction was observed in CHX-irrigating solutions showing the least antibacterial activity. The antibacterial activity was in the following sequence: NaOCl>iodine>CUR>triphala>CHX> PBS.

Forty-Minute Incubation

The mean CFU in specimens treated with PBS solution (control) was 200961145±114. The mean CFU in specimens treated with an iodine endodontic irrigating solution was 0. The CFU count was reduced significantly in comparison to the PBS control (p=0.002978). The mean CFU in specimens treated with NaOCl endodontic irrigating solution was 0. The CFU count was reduced significantly in comparison to the PBS control (p=0.003459). The mean CFU in specimens treated with CUR endodontic irrigating solution was 3356±345. The CFU count was reduced significantly in comparison to the PBS control (p=0.002941). The mean CFU in specimens treated with CHX endodontic irrigating solution was 2972233±873. The CFU count was reduced significantly in comparison to the PBS control (p=0.002927). The mean CFU in specimens treated with triphala endodontic irrigating solution was 361163±432. The CFU count was reduced significantly in comparison to the PBS control (p=0.000211). It was observed that a maximum reduction in CFU count was observed in NaOCl-treated specimens, showing maximum antibacterial activity against biofilm. It was followed by iodine and CUR. Triphala followed them. A minimum reduction was observed in CHX-irrigating solutions showing the least antibacterial activity. The antibacterial activity was in the following sequence: NaOCl>iodine>CUR>CHX>PBS (Table 2).

**Table 2:** Comparison of the effectiveness of various irrigation methods in sample B with PBS as the control. CFU: colony-forming units; SD: standard deviation; PBS: phosphate-buffered saline; CUR: curcumin; CHX: chlorhexidine; NaOCl: sodium hypochlorite

Variables	15 minutes (mean CFU±SD)	p-Value	40 minutes (mean CFU±SD)	p-Value
Iodine	380022±113	0.000311	0	0.002978
NaOCl	16671±108	0.000931	0	0.003459
CUR	360031±312	0.000342	3356±345	0.002941
CHX	18561143±416	0.000363	2972233±873	0.002927
Triphala	255555±142	0.000417	361163±432	0.002924
PBS	188531111±121	0.0001	200961145±114	0.0001

## Discussion

In the current study, two distinct in vitro biofilms have been investigated to determine how well they responded to various root canal irrigating chemicals [21-23]. The primary microbiological objectives of the mechanochemical preparation of root canals with infection are the full eradication of intracanal bacterial communities or, at the very least, the reduction of those organisms to levels favorable to periradicular healing of the tissues. According to Subramaniam et al., the periapical inflammation reaction is directly correlated with the presence of microorganisms [21].

When performing endodontic therapy, irrigating solutions must meet several criteria, one of which is an antibacterial activity that has been thoroughly studied regarding planktonic bacteria [24]. However, when broth as well as planktonic cultures are used for these assays, highly successful kills frequently result in results that don't match with clinical results [25]. It is now widely acknowledged that bacteria produced in biofilms express many phenotypes and frequently have rather different properties from those of bacteria cultivated in planktonic cultures [26].

When bacteria form biofilms, antimicrobial drugs can't get through as well because the bacteria's genetic and metabolic functions have changed, and their matrix is very complicated [27]. The colonizing organism then acquires defenses against unfavorable dietary as well as environmental factors [28]. The stronger resistance to antimicrobial drugs for a species with developed biofilm in contrasted with the same species growing planktonically may range from 100 to 1,000 times greater [29]. There is proof that oral bacterial biofilms are more resilient than planktonic cells to metronidazole, doxycycline, chlorhexidine, amoxicillin, and amine fluoride [30]. Costerton et al. say that this increased resistance to antibiotics may be due to changes in how bacteria use energy and how their genes are expressed that happen when they grow in a sessile state [30]. In a biofilm, it is also plausible to believe that microorganisms are less physically accessible. Regardless of the reason, reduced antimicrobial sensitivity renders standard antimicrobial chemotherapy useless in the management of biofilm-associated infections of the root canal [31]. To optimize and enhance the disinfection of root canals, numerous new irrigation methods, products, and distribution systems are now being introduced in endodontics [32]. Efficacy analysis in vivo is the gold standard for evaluating antibacterial remedies, but in vitro evaluation can also reveal important details regarding the strength and range of the individual compounds. The multispecies biofilm system utilized in the current study closely resembles in vivo biofilms, therefore enabling a standard evaluation of the antimicrobial drugs' effectiveness [31].

In this investigation, two distinct biofilms were chosen for resilience evaluation of antimicrobial irrigating solutions, one enriched in anaerobic organisms (sample A) and the other less diversified in anaerobic bacterium organisms and comprising exclusively Gram-positive strains of bacteria. Typically characterized by obligate anaerobic microorganisms, the primary infection of the root canal is polymicrobial [33]. In this study, the sample's bacterial makeup indicated that the infections in the tooth root canal were initial infections, which was consistent with the signs and symptoms the patients had. As can be seen from the data, sample B, which had a relatively low species diversity, demonstrated stronger resilience to the irrigating solutions across every test performed. Apparently, the reduced species count but a significantly greater count of specific microorganisms in sample B are responsible for this. As the standard of excellence in endodontic irrigation, NaOCl has completely eliminated all germs in sample A following 15 minutes of culture and in both of the specimens after 40 minutes. Iodine also completely eliminated all germs after 40 minutes of administration, indicating that it would be worth exploring using iodine as a potential endodontic irrigant. Iodine achieved total bacterial elimination after 40 minutes in both samples; however, it was less effective after 15 minutes. Our findings indicate that iodine solution is the most suitable alternative after the supremely effective NaOCl, although it requires longer contact times to generate the necessary and recognized broad-spectrum antibacterial properties, including in the case of biofilms. Furthermore, curcumin also showed significant results after NaOCl and iodine.

The scientific literature regarding CHX is highly divisive. NaOCl has been shown to be stronger in certain investigations [34], whereas CHX was discovered to be more effective in other research [35], and yet some studies indicated that there is no distinction [36]. In our study, NaOCl was much more effective than CHX at eliminating both biofilms over the two-time points that were taken into account. In our study, CUR also showed a significant reduction in CFUs in both sample A and sample B. However, it was observed that CUR was quite effective in sample A as compared to sample B. It showed that CUR can have better antibacterial activities against anaerobic bacteria. *C. longa*'s eradication of *E. faecalis*, one of the common bacteria linked to root canal therapy failure, showed encouraging results. *C. longa *could be an appropriate, cost-effective replacement for all previously used intracanal drugs because it has fewer negative effects and produces the least level of bacterial resistance among the species [1,32].

It was observed that the maximum reduction in CFU counts in sample A at 15- and 40-minute incubation was observed in NaOCl-treated specimens, showing maximum antibacterial activity against biofilm, followed by iodine irrigant solution. CFU and triphala were the ones who came after them. The reduction in CFU in CUR and triphala was comparable, with almost similar antibacterial activity against biofilm. A minimum reduction was observed in CHX-irrigating solutions showing the least antibacterial activity. The antibacterial activity was in the following sequence: NaOCl>Iodine>CUR=triphala>CHX>PBS. It was observed that a maximum reduction in CFU count in sample B at 15- and 40-minute incubation was observed in NaOCl-treated specimens, showing maximum antibacterial activity against biofilm. It was followed by iodine and CUR. Triphala followed them. A minimum reduction was observed in CHX-irrigating solutions showing the least antibacterial activity. The antibacterial activity was in the following sequence: NaOCl>Iodine>CUR>CHX>PBS.

In this study, triphala also showed a significant reduction in both sample A and sample B at both 15- and 40-minute incubation. However, it was more effective when used against anaerobic bacteria after 40 minutes of incubation. It has been traditionally used in Indian medicine to treat liver problems, headaches, and constipation. Triphala is harmless and has additional anti-inflammatory qualities and antioxidant capabilities. It is made up of substances with adequate physiologic actions when compared to numerous regularly used root canal irrigants [32].

The study has certain limitations such as, firstly, the study focused on a relatively small sample size of 60 patients with pulpitis. Expanding the sample size and including a more diverse patient population would enhance the generalizability of the findings and provide a more comprehensive understanding of these irrigants' performance in different clinical scenarios. Secondly, the in vitro nature of the biofilm experiments may not fully replicate the complex conditions found in actual root canals during endodontic therapy. In the case of endodontic therapy, root canals in vivo are subject to a dynamic environment influenced by blood circulation, tissue response, and other physiological factors that can affect treatment outcomes. An ex vivo model might not capture these complexities. Real-world factors, such as the presence of dentin, the anatomy of the root canal, and the presence of other substances, could influence the irrigants' effectiveness differently. Therefore, additional in vivo studies or clinical trials are warranted to validate the findings in a more clinically relevant context. Thirdly, the study primarily assessed the antibacterial activity of the irrigants but did not extensively evaluate their biocompatibility or potential side effects on the surrounding tissues.

## Conclusions

The antibacterial potency of each studied irrigant was significant, supporting their usage in endodontics. It was observed that NaOCl has the maximum antibacterial activity. Our findings indicate that iodine solution is the most suitable alternative after the supremely effective NaOCl, although it requires longer contact times to generate the necessary and recognized broad-spectrum antibacterial properties, including in the case of biofilms. Furthermore, curcumin also showed significant results after NaOCl and iodine. Our findings emphasize the relevance of studies into iodine analogs and curcumin analogs for use as endodontic irrigation by indicating that a solution including NaOCl, iodine, and CUR may provide the best effects.
